# A cross‐sectional study of sleep disturbance among middle‐aged cancer patients at Vietnam National Cancer Hospital

**DOI:** 10.1002/cnr2.2055

**Published:** 2024-04-05

**Authors:** Anh Tuan Pham, Mai Tuyet Do, Huong Thi Thanh Tran

**Affiliations:** ^1^ The Department of Optimal Clinical Care Vietnam National Cancer Hospital Hanoi Vietnam; ^2^ School of Preventive Medicine and Public Health Hanoi Medical University Hanoi Vietnam; ^3^ Department of Ethics & Medical Psychology Hanoi Medical University Hanoi VIetnam

**Keywords:** cancer patients, PSQI scale, sleep disorder, sleep disturbance, Vietnam

## Abstract

**Aim:**

Sleep disorders are common in cancer patients and have negative consequences for patient well‐being and treatment outcomes. This study aimed to investigate sleep quality and related factors in Vietnamese middle‐aged cancer patients.

**Methods:**

A cross‐sectional study was conducted on 246 middle‐aged in‐patient cancer patients at Vietnam National Cancer Hospital (VNCH) from 1/2021 to 7/2021. Sleep was measured by the Pittsburgh Sleep Quality Index (PSQI), with a cutoff of 5.

**Results:**

The results showed a male/female ratio of 0.85 with an average age of 52. The five most prevalent cancer types were breast, colorectal, lung, and esophagus‐stomach cancer, primarily in the late stage and treated with chemotherapy. The prevalence of sleep disturbances was 58.5%. The mean PSQI score was 7.5, with sleep duration and latency of 5.4 h and 1 h, respectively. Approximately 44% of participants reported poor sleep quality, nearly 9% had daytime dysfunction, and 10.6% used sleep medication. The multivariate logistic regression results indicate that people with depression were 8.89 times more likely to have poor sleep than those without depression (95% CI:2.63–28.27, *p* < .001).

**Conclusion:**

Sleep problems are common among middle‐aged people with cancer in Vietnam, especially individuals with depression. It is necessary to have more effective approaches to sleep management for cancer patients with limited resources.

## 1 INTRODUCTION

Cancer is a life‐threatening illness that has increasingly become a major burden on health, not only because it directly threatens human life but also because of its many negative indirect impacts on the quality of life of patients.^1^ According to GLOBOCAN, in 2020, there were 182 563 new cases of cancer in Vietnam, with nearly 123 000 deaths.^2^ A recent study identified that nearly 55% of Vietnamese cancer patients had distress, including both physical and psychosocial issues.^3^ In particular, cancer patients can experience sleep disturbances at any stage, from cancer diagnosis to post‐treatment.^4^ Even before cancer treatment, patients could face subjectively poor sleep.^5^, ^6^ Research has demonstrated that the prevalence of sleep disturbances in cancer patients may range from 26% to 57%, depending on studies and tools.^6^, ^7^, ^8^

Sleep health can be described as a multifaceted sleeping‐wakefulness schedule that is modified by personal, societal, and environmental factors that additionally foster both mental and physical health.^9^ According to the DSM‐V, insomnia disorder belongs to sleep–wake disorders, which are characterized by difficulty initiating or maintaining satisfactory sleep for at least three nights per week for three months or more.^10^ Insomnia in cancer patients is often caused by many factors, including biopsychosocial factors, particularly side effects of treatment or pain, leading to a remarkable decline in quality of life and treatment outcomes.^11^ An up‐to‐date review discovered a bidirectional relationship between sleep disorders and cancer, especially prevalent types of cancer such as breast cancer and lung cancer.^12^ Moreover, there is a body of evidence emphasizing various factors associated with sleep among people with cancer, such as marital status, employment, chemotherapy, late stage, and distressed physical signs.^11^ Regarding the consequences of lack of sleep, the study confirmed that sleep issues were associated with earlier progression and death, poor prognosis, and poor treatment response.^5^ Moreover, cancer patients with sleep disturbances are at higher risk of emotional disturbances, prolonged fatigue, and dependence on sedatives.^13^

In Vietnam, comprehensive care with the aim of improving the quality of life for cancer patients is increasingly focused, but scientific research on sleep disorders in this subject is still limited. A study in 2019 among breast cancer patients suggested a significant relationship between sleep issues and quality of life, in which the rate of sleep problems was 61%.^14^ A recent case–control study among colorectal adenomas in Vietnam emphasized that a lack of sleep is associated with a higher rate of colorectal cancer.^15^ The adapted three‐week psychoeducation for distressing symptoms was suggested to be effective for sleep management in Vietnamese cancer patients receiving chemotherapy.^16^ However, it is necessary to comprehensively assess the sleep status of cancer patients in Vietnam in many different contexts to obtain representative and scientific information on sleep management in comprehensive cancer care. The sleep was proven to relate to aging with higher risk and morbidity.^17^ Therefore, to investigate sleep problems in Vietnamese middle‐aged cancer patients and related factors, we conducted this study with two objectives: (1) to describe the sleep quality according to PSQI scale of middle‐aged cancer patients at Vietnam National Cancer Hospital (VNCH) in 2021, and (2) to evaluate the relationship between common demographic, cancer‐related factors and sleep disturbance in cancer patients at VNCH in 2021.

## 2 MATERIALS AND METHODS

### 2.1 Study design

A cross‐sectional study was conducted in 10 clinical in‐patient departments of VNCH, including four internal medicine departments, two surgery departments, two radiation departments, one general oncology department, and one palliative care department, from January to July 2021.

### 2.2 Study participants

The research participants were patients diagnosed and treated for cancer at 10 in‐patient departments of VNCH who met the following criteria:

- Inclusion criteria: (1) having confirmed cancer diagnosis and treatment at VNCH; (2) middle‐aged from 40 to 65 years old; (3) having the capacity to understand the questionnaires and interview; and (4) having no insomnia before cancer diagnosis.
- Exclusion criteria: having any severe physical or mental illness that can interfere with the study.

### 2.3 Sample size and sampling

Using the sample size calculation formula in cross‐sectional studies,^18^ the sample size *N* was determined with at type‐1 error of 5.0% (alpha = 0.05) and a 95% confidence level to achieve a statistical power of 80.0%. Utilizing a prevalence rate derived from prior study (*p* = .428, Hoang Thi Xuan Huong in 2019^8^) and aiming for a desired precision d of 0.07, it was found that a sample size of 192 was necessary. In fact, our study was conducted on 246 patients.

$$N=\frac{Z_{1-\frac{\alpha }{2}\left(1-P\right)P}^{2}}{d^{2}}$$

Three investigators were trained in research and data collection tools and then randomly approached cancer patients in each clinical in‐patient department and conducted face‐to‐face interviews. In particular, the interviewers randomly chose three departments among 10 available clinical departments on the list, then went to each department and randomly interviewed three patients in each in‐patient room. All interviewees and interviewers did not know about each other in advance, so the interviewers had to introduce themselves and research before inviting the patients to participate in the study. Eligible subjects were carefully informed about the study and signed a consent form before the interview. The data collection was finished when each department recruited at least 20 patients. There were 3 patients refused to participate because of lack of health to answer the questions. Therefore the agreement rate was 1.2% (3/249 patients).

### 2.4 Research variables and measurements

The general characteristics of the study subjects consisted of demographic information (including age, sex, marital status, education level, occupation, and medical insurance, and body mass index (BMI) classification according to the WHO^19^) and cancer‐related variables (including cancer site, disease stage, time from diagnosis in months, type of current treatment, and common physical symptoms).

The characteristics of participants' sleep were measured with the PSQI (Pittsburgh Sleep Quality Index) scale. This is a self‐assessment scale of sleep quality within the last month developed by the University of Pittsburgh, Pennsylvania, in 1988 by Buysse et al. A total score greater than 5 suggested a diagnostic sensitivity of 89.6% and a specificity of 86.5% in differentiating between good and poor sleepers.^20^ The scale consists of 19 items, creating 7 components to evaluate sleep quality, including subjectively perceived sleep quality, sleep latency, duration of sleep, habitual sleep efficiency (ratio of total sleep time and bed time), sleep disturbances, use of sleep medication (both prescription and over‐the‐counter), and daytime dysfunction. Each item is scored from 0 to 3 according to increasing impairment level. The interviewers were trained to ask the open questions to gather information from the patients according to the scale. The total score of the 7 components ranges from 0 to 21, where a higher score indicates a lower quality of sleep. The time to complete the scale is approximately 5–10 min. The scale is widely used in many countries around the world, including Vietnam.^21^, ^22^, ^23^, ^24^, ^25^ A systematic review of sleep disorders in cancer patients emphasized that the PSQI is the most commonly used subjective measure in this population.^26^ Prior study has validated the Vietnamese adaptation of PSQI scale as both a reliable and effective instrument for evaluating sleep disorders in Vietnamese patients and for community screening purposes.^27^ The analysis of internal consistency revealed that the Cronbach's alpha coefficient for this dataset was 0.84, demonstrating a good reliability. Besides, all participants were asked about the presence of the common physical symptoms including nausea or vomit, pain, and fatigue, constipation or diarrhea. These patients also were screened for depression and anxiety using the PHQ‐9 and GAD‐7 scales as a routine clinical practice at VNCH.

The Patient Health Questionnaire‐9 (PHQ‐9) is an extensively utilized self‐reporting instrument in mental health for the identification and quantification of depression severity. This tool encompasses nine components, each rated on a scale from 0 (indicating no occurrence) to 3 (reflecting near‐daily occurrence), basing on symptom frequency over the preceding fortnight. The total score, which can range from 0 to 27, is derived by totaling the individual scores of these nine components. The PHQ‐9**'**s validity and reliability have been confirmed within the Vietnamese context.^28^, ^29^ For our study, we adopted a threshold score of 8, informed by prior Vietnamese study, which demonstrated a sensitivity of 87.0% and a specificity of 82.4% at this cutoff.^28^

The Generalized Anxiety Disorder 7 (GAD‐7) is a widely used self‐report assessment tool designed to screen and measure the severity of generalized anxiety disorder. The GAD‐7 consists of seven questions, each assessing a different aspect of anxiety symptoms over the past two weeks. Respondents rate the frequency of their symptoms on a scale from 0 (not at all) to 3 (nearly every day) for each question. The total score ranges from 0 to 21, with higher scores indicating more severe anxiety symptoms. The validity and reliability of this scale have been assessed within the Vietnamese population including cancer patients.^3^, ^30^ This study used a cut‐off level of 10, with sensitivity (89%) and specificity (82%).^31^

### 2.5 Statistical analysis

Data were entered and analyzed by SPSS 20.0 software. Descriptive statistical metrics including frequency, percentage, mean, and standard deviation (SD) were employed. The study utilized a boxplot to illustrate the median and interquartile range of the total PSQI score based on the site of the cancer. A logistic regression model was employed to estimate odds ratios and 95% confidence intervals, thereby elucidating the relationship between associated factors and sleep disturbances. The dependent variable was a PSQI score of 5 or greater, indicative of sleep disorders, while the independent variables included age, gender, BMI classification, time since diagnosis, cancer site, disease stage, depression, anxiety, marital status, and presenting symptoms. The level of statistical significance was established at a *p*‐value below 0.05.

### 2.6 Research ethics

The study was approved by the VNCH Scientific Council in 2021 and followed research ethical standards. All participants were fully informed about the research and signed a consent form before participating in the study.

## 3 RESULTS

Table 1 shows that the male/female ratio of the study subjects was 0.85, and the average age was approximately 52. Most of the participants were married and had a secondary‐high school education. The five most common cancer sites were breast, colorectal, lung, and esophagus‐stomach cancer, mainly in late stage III‐IV (60.9%) and treated with chemotherapy. The most common physical symptoms were fatigue and pain.

**TABLE 1:** The characteristics of the cancer patient (*n* = 246).

Characteristics	*n* (%)
Demographic characteristics	
*Age (year)*	
Mean (SD)	51.6 (9.78)
*Gender*	
Male	113 (44.9)
Female	133 (54.1)
*Marital status*	
Married	222 (90.2)
Single	10 (4.1)
Divorced	4 (1.6)
Widowed	10 (4.1)
*BMI classification*	
Underweight (< 18.5)	53 (21.5)
Normal (18.5‐ < 25)	184 (74.8)
Overweight (≥ 25)	9 (3.7)
*Occupation*	
Farmer	126 (51.2)
Worker	22 (8.9)
Public employee	33 (13.4)
Business	26 (10,6)
Other	39 (15.9)
*Educational level*	
Primary school	25 (10.2)
Secondary school	113 (45.9)
High school	71 (28.9)
College or higher	37 (15.0)
*Having medical insurance*	
Yes	239 (97.2)
No	7 (2.8)
Cancer‐related and treatment characteristics	
*Cancer site*	
Breast	55 (22.4)
Colorectal	48 (19.5)
Lung	28 (11.4)
Esophagus	28 (11.4)
Stomach	22 (8.9)
Other	65 (26.4)
*Disease stage*	
I	25 (10.2)
II	71 (28.9)
III	99 (40.2)
IV	51 (20.7)
*Time since diagnosis (months)*	
Mean (SD)	13.0 (26.07)
*Current treatments*	
Chemotherapy	125 (50.8)
Radiotherapy	48 (19.5)
Surgery	48 (19.5)
Other	25 (10.2)
*Common physical symptoms*	
Nausea/Vomit	59 (24.0)
Pain	103 (41.9)
Fatigue	122 (49.6)
Constipation/Diarrhea	78 (31.7)
Mental health symptom characteristics	**Mean (SD)**
PHQ‐9 depression score	4.7 (4.4)
GAD‐7 anxiety score	3.3 (4.3)

According to the PSQI scale, 58.5% of the study subjects had sleep disturbances. For more detailed information, the scores were divided into four categories^32^ based on the distribution of PSQI global score in primary validation study of Buyse.^20^ In particular, 26.4% of participants had sleep problems at moderate to severe levels (Table 2).

**TABLE 2:** The severity of sleep disturbance in study subjects (*n* = 246).

Sleep disturbance	*n*	%
Normal (<5 score)	102	41.5
Mild (5–9 score)	79	32.1
Moderate (10–14 score)	53	21.5
Severe (>15 score)	12	4.9

Table 3 demonstrates the different components of sleep disorders, with an average PSQI score of 7.5. The mean sleep duration was less than 5.5 h per night, and the sleep latency was nearly 1 h. Approximately 9% of subjects felt the negative impact of insomnia on daytime functioning; and more than 10% of people used medication to support sleep.

**TABLE 3:** The characteristics of sleep disorders in study subjects (*n* = 246).

Components of sleep quality on PSQI	Mean (SD)
Average sleep time (hours)	5.4 (1.7)
Average time to fall asleep (hours)	0.9 (1.3)
Having daytime dysfunction due to poor sleep (*n* %)	22 (8.9%)
Use of sleeping medication (*n* %)	26 (10.6%)
Average PSQI score	7.5 (4.2)
Components of PSQI	
Sleep quality	1.4 (8.8)
Sleep latency	1.7 (1.1)
Duration of sleep	1.7 (1.1)
Sleep efficiency	1.2 (1.3)
Sleep disturbance	1.1 (0.4)
Use of sleep medication	0.2 (0.7)
Daytime dysfunction	1.4 (0.9)

**FIGURE 1:**
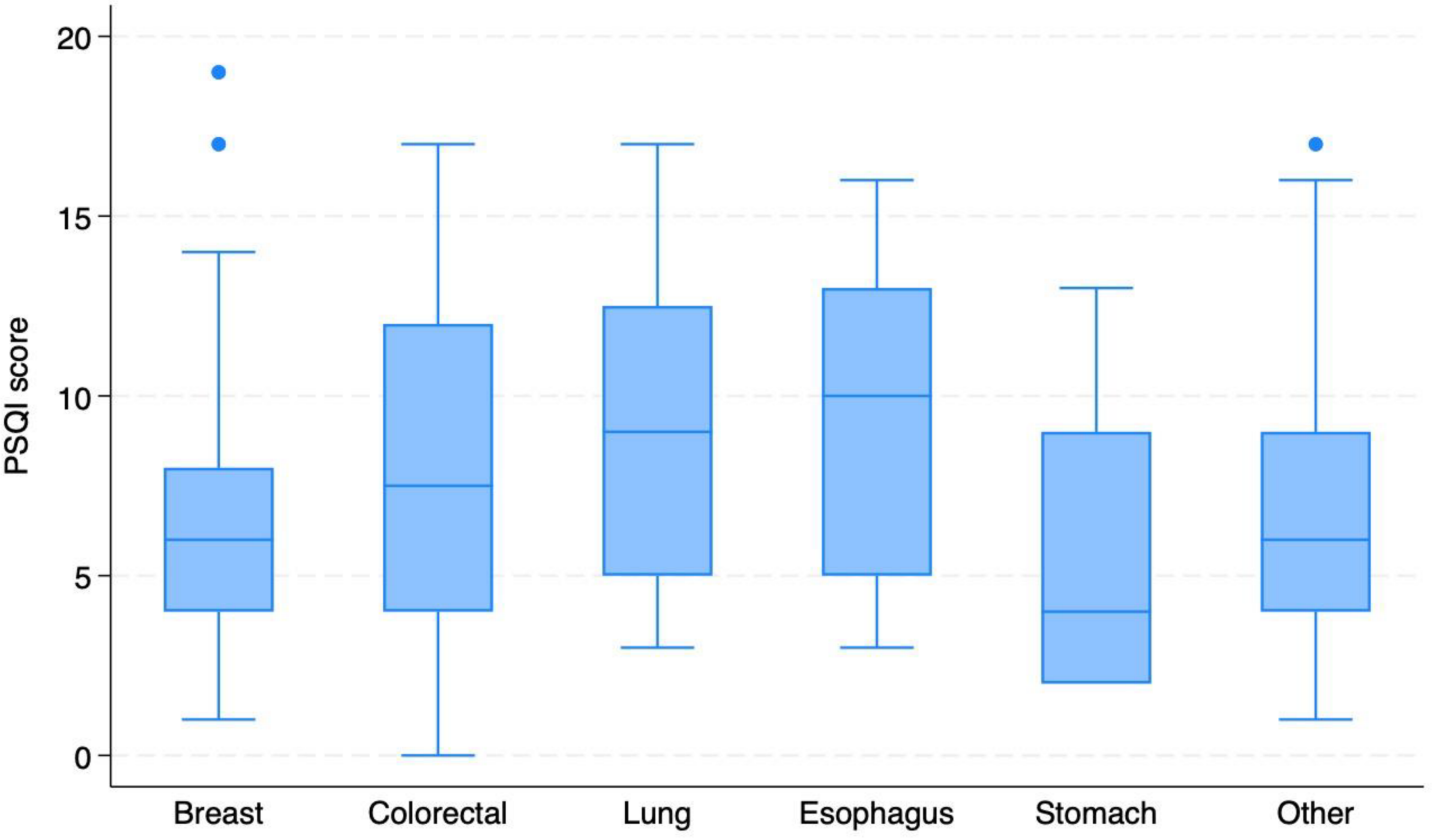
Total PSQI score by cancer site.

Figure 1 presents boxplot results classifying total PSQI scores by cancer site. The cancer with the highest sleep disorders median is esophageal cancer (median 10, IQR: 5–13), followed by lung cancer (median 9, IQR: 5–12.5) and colorectal cancer (median 7.5; IQR: 4–12).

Table 4 displays the multivariate logistic regression results, indicating that individuals with depressive symptoms were 8.89 times more likely to have poor sleep than their non‐depressed counterparts (95% CI: 2.63–28.27). Lung and esophageal cancers were associated with higher odds ratios, 2.58 and 2.57 respectively, although these findings did not achieve statistical significance.

**TABLE 4:** Multivariable logistic regression of factors associated to sleep problems (*n* = 246).

Characteristics	Total	Poor sleep (%)	OR	95% CI	*p* value
Gender					
Male	113	66 (58.4)	1.00		
Female	133	78 (58.6)	1.15	(0.53–2.49)	0.716
BMI classification					
Underweight	53	37 (69.8)	1.76	(0.82–3.79)	0.147
Normal	184	99 (53.8)	1.00		
Overweight	9	8 (88.9)	4.52	(0.45–45.27)	0.199
Age (years)			1.00	(0.97–1.04)	0.864
Marital status					
Married	138	131 (59.0)	1.00		
Single/Divorced/Widowed	108	13 (54.2)	0.80	(0.29–2.21)	0.663
Educational level					
<High school	138	87 (63.0)	1.00		
≥High school	108	57 (52.8)	0.90	(0.48–1.69)	0.743
Time since diagnosis (months)			1.14	(0.96–1.37)	0.145
Depression (PHQ‐9 ≥ 8)	52	47 (90.4)	**8.89**	**(2.72–29.01)**	**0.000**
Anxiety (GAD‐7 ≥ 10)	25	20 (80.0)	0.92	(0.23–3.72)	0.910
Treatments					
Surgery	48	27 (56.2)	0.54	(0.14–2.04)	0.363
Radiation therapy	48	27 (56.2)	0.60	(0.18–2.03)	0.413
Chemotherapy	125	71 (56.8)	0.54	(0.18–1.63)	0.273
Others	25	19 (76.0)	1.00		
Disease stage					
I	25	13 (52.0)	1.00		
II	71	36 (50.7)	0.93	(0.32–2.68)	0.886
III	99	63 (63.6)	1.34	(0.48–3.78)	0.575
IV	51	32 (62.7)	1.01	(0.31–3.32)	0.982
Cancer site					
Breast	55	28 (50.9)	1.00		
Colorectal	48	32 (66.7)	2.12	(0.72–6.24)	0.173
Lung	28	19 (67.9)	1.48	(0.41–5.33)	0.550
Esophagus	28	20 (71.4)	2.67	(0.69–10.29)	0.154
Stomach	22	9 (40.9)	0.88	(0.20–3.90)	0.869
Other	65	36 (55.4)	1.25	(0.50–3.13)	0.639
Pain	103	68 (66.0)	1.06	(0.56–2.01)	0.854
Nausea/Vomit	59	40 (67.8)	1.21	(0.56–2.58)	0.630
Fatigue	122	78 (63.9)	1.43	(0.76–2.68)	0.265
Constipation/Diarrhea	78	51 (65.4)	1.09	(0.53–2.23)	0.813

## 4 DISCUSSION

This study on the sleep patterns of cancer patients at VNCH recruited 246 middle‐aged participants with a male/female ratio of 0.85 and an average age of 52. The five most common cancers were breast, colorectal, lung, and esophagus‐stomach cancer, mainly in late stages III‐IV. More than 40% of patients were treated with chemotherapy and had fatigue and pain.

According to the PSQI scale, 58.6% of the study subjects had sleep problems, and the rate of moderate‐to‐severe insomnia was approximately 26%. This is in line with a recent systematic review about sleep trouble in cancer settings that exhibited an overall figure of 60.7%.^33^ Our study also presented a mean global PSQI score of 7.5, which is equivalent to a mild level of sleep disorder. In particular, we found that the sleep duration was less than an average of 5.5 h, and the time of falling asleep was roughly **1** h. This result is consistent with Pai's study in India showing that 57.5% of cancer patients have sleep disorders according to the PSQI scale, in which the average PSQI score is 6.57 ± 3.52, nearly 40% reported sleeping less than 6 h per night and 32.6% were affected by daytime activities due to lack of sleep.^34^ Savard's study longitudinally monitoring insomnia symptoms in people with malignancies also recorded a rate of 37% to 66% depending on disease diagnosis, treatment method, and follow‐up time.^4^ A study applying the PSQI among 179 individuals with lung, breast, prostate, and brain cancer indicated that the rate of sleep problems was 57%, which was related to and significantly higher than mood problems.^7^ In Vietnam, research by Hoang Thi Xuan Huong among 213 cancer patients also recorded the rate of insomnia as 42.8%, in which the average sleep time was approximately 5–6 h.^8^ The higher rate of sleep issues in our study can be explained by the fact that the majority of our study population was less than 60 years old, which has been proven to be related to lower‐quality sleep compared to the group of 60 years old and above.^25^ The study result is also consistent with a previous study in cancer patients in Vietnam that identified that the age group of 46–60 years had a higher rate of bad sleep than younger groups.^35^ Furthermore, most participants in our study were in late‐stage cancer and undergoing chemotherapy, which are factors that may have a more negative influence on sleep quality.^13^ The application of other measures, such as the distress thermometer, also showed a higher proportion of poor sleep in oncology, from 50% **to** 60%.^3^, ^36^

In this study, 44% of participants self‐reported poor sleep quality, nearly 9% of subjects had impaired functioning, and approximately 10% used medication for sleep. This is in accordance with the findings of previous studies that identified that the prevalence of sleep problems in oncology participants was approximately 30% to 50%, depending on subjective or objective measures.^6^ Subjective poor sleep was observed to be related to worse cancer outcomes.^5^ When sleep quality is poorer, patients may experience a greater impact on their daily lives, cancer treatment satisfaction, and quality of care. A case–control study in a Vietnamese cancer population also revealed that inadequate sleep time may be negatively correlated with a worse prognosis for cancer.^15^ In addition, our finding is suitable for the fact that the vast majority of psychotropic drugs prescribed to cancer patients have been for sleep aids, much higher than for other reasons such as psychological problems or nausea.^11^ The combination of sedatives may worsen daytime fatigue and increase the risk of side effects and drug interactions during cancer treatment. Currently, in Vietnam, psychosocial interventions to improve sleep in cancer patients are still limited, so the abuse of sleeping pills in this population may be a common problem. Thus, many studies have shown the critical prevalence of sleep disorders with significant impairment in cancer patients, which is the basis for more effective sleep interventions to improve the quality of life of this vulnerable subject.

In terms of sleep‐related factors, our study identified that sleep disturbances in cancer patients were significantly associated with depressive symptoms. The worse sleep was noticed more commonly in patients with esophagus cancer, lung cancer, and colorectal cancer. No association was found between sleep quality and demographic factors, other cancer‐related factors, pain or current treatment. This is in line with a study by Akman in 314 Turkish cancer patients, which showed no remarkable relationship between PSQI score and gender, marital status, cancer stage, or chemotherapy but metastasis conditions with lower scores.^23^ Our findings are consistent from those of previous studies reporting that lung cancer was related to high sleep complaints, but not the same with breast cancer patients.^11^ This difference may be due to the small sample size in our study, which is not representative of multiple types of cancer relating to different rates of sleep quality. However, even epidemiological data on 1 500 000 patients among 13 nations did not conclude a valid statement about sleep and cancer.^37^ Therefore, more research is needed to delve into the mechanism and sleep symptoms in each type of cancer to obtain more accurate results about this relationship, if any. In addition, many studies have pointed out the significance of three main insomnia‐related factors, including predisposing factors (i.e., female sex and aging), precipitating factors (i.e., cancer symptoms and treatment), and perpetuating factors (i.e., unhealthy sleep habits and attitudes).^11^, ^13^ All of these factors may simultaneously contribute to poor sleep quality during treatment, especially depression, which is more common and more severe in late‐stage patients, as in our study population. The review about depression and insomnia in cancer revealed clinical significance with multiple underlying biological and psychological mechanisms.^38^ The study of Sateia also explored the significant relevance between poor sleep and common physical and psychological symptoms.^39^ A study on distress among Vietnamese cancer patients in 2020 also found that insomnia was statistically related to psychological distress in patients with cancer.^3^

In general, in clinical care for people with malignancies, clinicians should pay more attention to managing sleep impairments for patients with depression, helping to enhance quality of life and patient satisfaction as well as treatment outcomes in general.

### 4.1 Study limitations

This is the first descriptive study on sleep disorders conducted on a diverse sample size in many clinical departments at VNCH. Study subjects were randomly approached during inpatient treatment, including radiation therapy, chemotherapy, and surgery. Therefore, the study population included various types of cancer and different treatments. However, the study still has certain limitations. First, the study had a limited sample size that was recruited in a short time, which was not representative of the cancer population in Vietnam with many different personal and social characteristics. Second, the demographic factors and physical symptoms surveyed in this research were inadequate. For instance, the majority of participants were in stages 3 and 4 of cancer which may cause bias so the results did not fully demonstrate the sleep quality of the whole population of people living with cancer or not detect the factors affecting sleep quality in cancer patients. Therefore, it is necessary to conduct further studies with larger and targeted sample sizes in multiple centers nationwide to more accurately investigate the sleep issues of cancer patients, which is the basis for developing effective sleep interventions for this vulnerable population, especially in scarce‐resource countries, such as Vietnam.

## 5 CONCLUSIONS

Sleep disorders are common in cancer patients and can reduce patients' quality of life and ability to function during the day. The study findings showed nearly 60% of cases with low sleep quality during cancer treatment, especially at moderate and severe levels. Furthermore, the results revealed a statistically significant association between sleep disturbance and depression in cancer adults. More studies with more representative research populations and more detailed evaluations of sleep disorders and management in cancer patients are needed to contribute to comprehensive oncology care in Vietnam.

## AUTHOR CONTRIBUTIONS

**Anh Tuan Pham:** Conceptualization (lead); formal analysis (equal); investigation (supporting); methodology (equal); project administration (equal); resources (supporting); supervision (supporting); writing – original draft (equal); writing – review and editing (equal). **Mai Tuyet Do:** Conceptualization (equal); formal analysis (equal); funding acquisition (equal); investigation (equal); methodology (equal); project administration (equal); writing – original draft (equal); writing – review and editing (equal). **Huong Thi Thanh Tran:** Conceptualization (equal); funding acquisition (equal); investigation (supporting); project administration (equal); resources (equal); supervision (equal); writing – original draft (equal); writing – review and editing (equal).

## FUNDING INFORMATION

This research received no external funding.

## CONFLICT OF INTEREST STATEMENT

The authors declare no conflicts of interest.

## INSTITUTIONAL REVIEW BOARD STATEMENT

The study was conducted in accordance with the Declaration of Helsinki and approved by the Vietnam National Cancer Hospital's Scientific Council in 2021.

## INFORMED CONSENT STATEMENT

Informed consent was obtained from all subjects involved in the study.

## Data Availability

The data that support the findings of this study are available on request from the corresponding author. The data are not publicly available due to privacy or ethical restrictions.
